# Supplementary material for: Multi‐dataset identification of innovative feature genes and molecular mechanisms in keratoconus
Source: J Cell Mol Med. 2024 Sep 19;28(18):e70079. doi: 10.1111/jcmm.70079 (PMC11412914; doi:10.1111/jcmm.70079)
Supplement: Supplementary file 2 — Material S2. [file JCMM-28-e70079-s002.zip › 7.GSEA analysis/6.SNORA74B/KEGG_MELANOGENESIS.html]

Details for gene set KEGG\_MELANOGENESIS[GSEA]

|  || Dataset | SNORA74B.group.cls#h\_versus\_l |
| Phenotype | group.cls#h\_versus\_l |
| Upregulated in class | l |
| GeneSet | KEGG\_MELANOGENESIS |
| Enrichment Score (ES) | -0.42878124 |
| Normalized Enrichment Score (NES) | -1.6201415 |
| Nominal p-value | 0.01764706 |
| FDR q-value | 0.26076353 |
| FWER p-Value | 0.579 |
Table: GSEA Results Summary

  

Fig 1: Enrichment plot: KEGG\_MELANOGENESIS      
 Profile of the Running ES Score & Positions of GeneSet Members on the Rank Ordered List

  

| SYMBOL | TITLE | RANK IN GENE LIST | RANK METRIC SCORE | RUNNING ES | CORE ENRICHMENT || 1 | CAMK2B | na | 141 | 0.835 | 0.0433 | No |
| 2 | MC1R | na | 1641 | 0.403 | 0.0229 | No |
| 3 | PRKCG | na | 2539 | 0.330 | 0.0157 | No |
| 4 | ADCY2 | na | 2866 | 0.309 | 0.0239 | No |
| 5 | CAMK2A | na | 3838 | 0.265 | 0.0109 | No |
| 6 | ADCY5 | na | 6025 | 0.192 | -0.0414 | No |
| 7 | ADCY7 | na | 6070 | 0.190 | -0.0319 | No |
| 8 | ASIP | na | 6144 | 0.188 | -0.0233 | No |
| 9 | CALML6 | na | 7151 | 0.158 | -0.0434 | No |
| 10 | ADCY4 | na | 7191 | 0.157 | -0.0356 | No |
| 11 | GNAI3 | na | 7691 | 0.144 | -0.0419 | No |
| 12 | WNT16 | na | 8300 | 0.129 | -0.0521 | No |
| 13 | CALML5 | na | 8469 | 0.125 | -0.0498 | No |
| 14 | WNT10B | na | 8944 | 0.115 | -0.0570 | No |
| 15 | GNAO1 | na | 9178 | 0.110 | -0.0575 | No |
| 16 | PLCB3 | na | 9334 | 0.106 | -0.0560 | No |
| 17 | PLCB2 | na | 9573 | 0.101 | -0.0571 | No |
| 18 | KRAS | na | 9766 | 0.097 | -0.0571 | No |
| 19 | WNT5A | na | 10441 | 0.084 | -0.0718 | No |
| 20 | CREBBP | na | 10525 | 0.083 | -0.0695 | No |
| 21 | ADCY9 | na | 10911 | 0.076 | -0.0763 | No |
| 22 | ADCY1 | na | 11165 | 0.072 | -0.0795 | No |
| 23 | EDNRB | na | 11740 | 0.064 | -0.0924 | No |
| 24 | GNAQ | na | 11760 | 0.063 | -0.0894 | No |
| 25 | CAMK2G | na | 11900 | 0.062 | -0.0899 | No |
| 26 | WNT8A | na | 12290 | 0.056 | -0.0980 | No |
| 27 | CALM2 | na | 12514 | 0.053 | -0.1014 | No |
| 28 | WNT3 | na | 12537 | 0.053 | -0.0991 | No |
| 29 | ADCY6 | na | 12561 | 0.052 | -0.0967 | No |
| 30 | TCF7L2 | na | 12663 | 0.051 | -0.0968 | No |
| 31 | WNT2 | na | 14134 | 0.034 | -0.1373 | No |
| 32 | FZD1 | na | 14343 | 0.032 | -0.1415 | No |
| 33 | GNAI2 | na | 14445 | 0.031 | -0.1426 | No |
| 34 | MAP2K2 | na | 14904 | 0.025 | -0.1544 | No |
| 35 | CALML3 | na | 15332 | 0.021 | -0.1655 | No |
| 36 | FZD5 | na | 15466 | 0.020 | -0.1683 | No |
| 37 | CREB3 | na | 15657 | 0.018 | -0.1727 | No |
| 38 | CREB3L1 | na | 15845 | 0.016 | -0.1772 | No |
| 39 | FZD6 | na | 16365 | 0.011 | -0.1916 | No |
| 40 | MAPK3 | na | 16753 | 0.007 | -0.2024 | No |
| 41 | PLCB4 | na | 16838 | 0.006 | -0.2044 | No |
| 42 | CTNNB1 | na | 17783 | -0.003 | -0.2315 | No |
| 43 | GSK3B | na | 17871 | -0.004 | -0.2338 | No |
| 44 | FZD9 | na | 18101 | -0.007 | -0.2400 | No |
| 45 | WNT4 | na | 18195 | -0.008 | -0.2422 | No |
| 46 | CREB3L3 | na | 18368 | -0.010 | -0.2466 | No |
| 47 | WNT3A | na | 18601 | -0.013 | -0.2526 | No |
| 48 | ADCY8 | na | 18678 | -0.014 | -0.2540 | No |
| 49 | FZD3 | na | 18707 | -0.014 | -0.2540 | No |
| 50 | HRAS | na | 19699 | -0.025 | -0.2812 | No |
| 51 | WNT9A | na | 20087 | -0.029 | -0.2907 | No |
| 52 | GNAS | na | 20567 | -0.035 | -0.3025 | No |
| 53 | MAPK1 | na | 20606 | -0.035 | -0.3016 | No |
| 54 | DVL3 | na | 20946 | -0.039 | -0.3092 | No |
| 55 | RAF1 | na | 21074 | -0.041 | -0.3105 | No |
| 56 | PRKX | na | 21076 | -0.041 | -0.3083 | No |
| 57 | CREB3L4 | na | 21237 | -0.043 | -0.3105 | No |
| 58 | DVL1 | na | 21883 | -0.050 | -0.3262 | No |
| 59 | CREB3L2 | na | 22159 | -0.054 | -0.3311 | No |
| 60 | PRKCA | na | 22239 | -0.055 | -0.3303 | No |
| 61 | CALM1 | na | 22340 | -0.056 | -0.3300 | No |
| 62 | PRKACA | na | 22659 | -0.060 | -0.3358 | No |
| 63 | KIT | na | 23499 | -0.073 | -0.3559 | No |
| 64 | FZD4 | na | 23557 | -0.074 | -0.3533 | No |
| 65 | CALM3 | na | 23658 | -0.075 | -0.3519 | No |
| 66 | WNT2B | na | 25314 | -0.104 | -0.3938 | No |
| 67 | EP300 | na | 25385 | -0.105 | -0.3899 | No |
| 68 | FZD8 | na | 25688 | -0.111 | -0.3923 | No |
| 69 | KITLG | na | 25692 | -0.111 | -0.3861 | No |
| 70 | CREB1 | na | 25963 | -0.116 | -0.3874 | No |
| 71 | DVL2 | na | 26618 | -0.130 | -0.3988 | No |
| 72 | ADCY3 | na | 26777 | -0.134 | -0.3958 | No |
| 73 | WNT10A | na | 26979 | -0.139 | -0.3937 | No |
| 74 | TCF7L1 | na | 27185 | -0.144 | -0.3914 | No |
| 75 | FZD10 | na | 27856 | -0.161 | -0.4017 | No |
| 76 | NRAS | na | 27969 | -0.164 | -0.3956 | No |
| 77 | GNAI1 | na | 28399 | -0.176 | -0.3980 | No |
| 78 | TCF7 | na | 28810 | -0.189 | -0.3991 | No |
| 79 | WNT7B | na | 28850 | -0.190 | -0.3894 | No |
| 80 | WNT8B | na | 29836 | -0.219 | -0.4054 | No |
| 81 | WNT9B | na | 30647 | -0.250 | -0.4146 | Yes |
| 82 | EDN1 | na | 31118 | -0.274 | -0.4126 | Yes |
| 83 | MAP2K1 | na | 31129 | -0.275 | -0.3973 | Yes |
| 84 | WNT7A | na | 31601 | -0.301 | -0.3938 | Yes |
| 85 | POMC | na | 31671 | -0.305 | -0.3785 | Yes |
| 86 | WNT1 | na | 32208 | -0.335 | -0.3750 | Yes |
| 87 | WNT6 | na | 32236 | -0.336 | -0.3567 | Yes |
| 88 | PRKACB | na | 32349 | -0.344 | -0.3404 | Yes |
| 89 | FZD2 | na | 32364 | -0.345 | -0.3212 | Yes |
| 90 | WNT5B | na | 32480 | -0.353 | -0.3045 | Yes |
| 91 | DCT | na | 32497 | -0.354 | -0.2848 | Yes |
| 92 | CAMK2D | na | 32655 | -0.364 | -0.2687 | Yes |
| 93 | PRKACG | na | 32698 | -0.368 | -0.2490 | Yes |
| 94 | PLCB1 | na | 32854 | -0.381 | -0.2319 | Yes |
| 95 | FZD7 | na | 33265 | -0.421 | -0.2199 | Yes |
| 96 | PRKCB | na | 33882 | -0.508 | -0.2088 | Yes |
| 97 | LEF1 | na | 34351 | -0.633 | -0.1864 | Yes |
| 98 | WNT11 | na | 34430 | -0.665 | -0.1509 | Yes |
| 99 | TYRP1 | na | 34624 | -0.831 | -0.1093 | Yes |
| 100 | MITF | na | 34665 | -0.895 | -0.0596 | Yes |
| 101 | TYR | na | 34712 | -1.082 | 0.0005 | Yes |
Table: GSEA details [plain text format]

  

Fig 2: KEGG\_MELANOGENESIS      
 Blue-Pink O' Gram in the Space of the Analyzed GeneSet

  

Fig 3: KEGG\_MELANOGENESIS: Random ES distribution      
 Gene set null distribution of ES for **KEGG\_MELANOGENESIS**
